# 3-D Design and Simulation of a Piezoelectric Micropump

**DOI:** 10.3390/mi10040259

**Published:** 2019-04-18

**Authors:** Seyed Amir Fouad Farshchi Yazdi, Alberto Corigliano, Raffaele Ardito

**Affiliations:** MEMS Modelling and Design Group, Department of Civil and Environmental Engineering, Politecnico di Milano, 20133 Milan, Italy; seyedamir.farshchi@polimi.it (S.A.F.F.Y.); raffaele.ardito@polimi.it (R.A.)

**Keywords:** piezoelectric material, multiphysics simulation, finite element method (FEM), fluid–structure interaction (FSI), micro electromechanical systems (MEMS)

## Abstract

The objective of this paper is to carefully study the performances of a new piezoelectric micropump that could be used, e.g., for drug delivery or micro-cooling systems. The proposed micropump is characterized by silicon diaphragms, with a piezoelectric actuation at a 60 V input voltage, and by two passive valves for flow input and output. By means of a 3-D Finite Element (FE) model, the fluid dynamic response during different stages of the working cycle is investigated, together with the fluid–structure interaction. The maximum predicted outflow is 1.62 μL min${}^{-1}$, obtained at 10 Hz working frequency. The computational model enables the optimization of geometrical features, with the goal to improve the pumping efficiency: The outflow is increased until 2.5 μL min${}^{-1}$.

## 1. Introduction

Pumps are devices that provide momentum to transfer fluids and can be integrated in Micro Electromechanical Systems (MEMS) to develop so-called micro-fluidic systems [1]. As an example, micropumps play an important role in biomedical and drug delivery systems: The micro-dosing feature in such devices has improved the effectiveness of the treatment because the concentration of the drug in the patient’s body has been kept constant as well as because toxicity has been prevented [2].

Several actuation systems have been investigated in the literature [3], with particular attention to electrostatic forces and piezoelectric materials. The former case, e.g., studied in Reference [4], is connected to some drawbacks related to the high actuation voltage and the possible occurrence of pull-in instability [5]; on the other hand, piezoelectric actuation has been widely utilized based on advantages such as the small size, low power consumptions, no electromagnetic interference, and an insensitivity of fluid viscosity [6] even though the maximum power density of the piezoelectric materials is dependent on the working frequency [7]. Among different materials with a piezoelectric effect, lead zirconate titanate (PZT) ceramics demonstrated optimal performances in view of the large deflection that can be induced in the pump diaphragm, as recently shown by Cazorla et al. [8]. While Nisar et al. have reported different types of micropumps fabricated for biomedical applications [9], there are some recent studies about the design and modelling of piezoelectric micropumps. Revathi and Padmanabhan designed a valveless micropump with a composite piezoelectric actuator which showed the maximum outflow at an aspect ratio of 15 for a nozzle/diffuser [10]. Sateesh et al. [11] designed and modelled a piezoelectrically actuated micropump with PZT-5h and Polydimethylsiloxane (PDMS) with an outflow rate of 0.029 μL s${}^{-1}$.

In any case, it has been clearly established that, in addition to the actuator, the presence of valves in the device can affect the performance. Despite the fact that the valveless micropumps have the benefit of no risk of wear and fatigue of the valves, it is hard to control the flow to the desired direction at inlet and outlet, causing energy loss and liquid reflux. Consequently, in order to improve the control of the fluid flow, mechanical valves (either active or passive) must be introduced in the system. The main novelty of the present paper is the comprehensive study of a complete pumping system, composed of a piezoelectric actuator and passive valves, using three-dimensional modelling and a simulation of the device performing a complete cycle of pumping with a consideration for the two-way fluid–structure interaction. An innovative layout is considered, starting from a patent [12] that was originally proposed for electrostatic actuation. The purpose of the present paper is to assess the behavior of the new device and to propose some slight modifications in order to improve the performances.

The paper is organized as follows. After the introduction, the layout of the device is shortly discussed. The subsequent section contains a thorough description of the computational model which then leads to some preliminary results. Then, we consider some modifications of the geometrical features of the pump, with the purpose of optimizing its performances.

## 2. Description of the Proposed Layout

In most cases, a pumping device, composed of a micropump and valves, is of complex realization in view of the difficult integration of the different components of the actuating system on a limited number of wafers. Moreover, the integrated valves may present specific issues related to a lack of tightness, which is the cause of a leakage and backflow.

**Figure 1:**
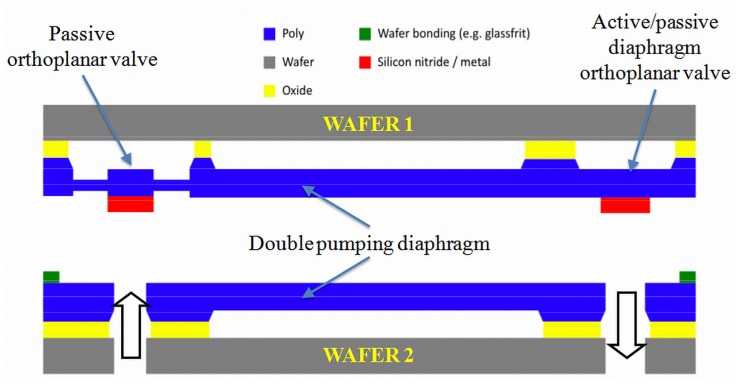
A schematic view of the micropump proposed in Reference [12] and adopted in the present paper with the addition of a piezoelectric actuation.

In order to overcome the aforementioned limits, an innovative device based on the use of two wafers has been recently conceived and patented [12]. In spite of the fact that the invention refers to an electrostatic actuation, the main advantage of using just two wafers for an integrated design can be exploited also for the piezoelectric case. According to the invention, the pumping device comprises (i) a pumping chamber, realized between two silicon wafers bonded to each other; (ii) an inlet valve, with a shutter element in correspondence to the connection with the external reservoir; and (iii) an outlet valve, with a shutter element on the external microfluidic circuit. As shown in Figure 1, when the inlet valve is in the open configuration, the shutter is housed by a recess that is fluidly coupled with the pumping chamber by means of an inlet channel. On the other hand, the outlet shutter is located in a recess that is fluidly decoupled with respect to the pumping chamber. The described configuration of the inlet and outlet valves allows the direction of the processed flow to be controlled in a completely passive way. More precisely, the inlet and outlet valves do not require dedicated actuators, and so, the structure is generally simplified for the benefit of both the overall dimensions and the manufacturing costs. For instance, the micropump, as above defined, may be made from just two semiconductor wafers joined together. Moreover, the micropump control is simplified because it does not have to take into account the synchronization of the valves. Dedicated actuators for the valves, in particular for the output valve, may optionally be provided if specific circumstances make this advisable. However, the micropump is still fully operative even with purely passive valves. The inlet valve and the outlet valve are of the orthoplanar type. The sealing is guaranteed by the presence of an initial stress state that can be easily achieved during the bonding phase by introducing a couple of thick elements on the shutters: If the thickness of such elements is larger than the bonding layer, the orthoplanar valves are forced to close the holes. It is worth noting that the layout of the device allows for the introduction of two opposite pumping diaphragms, with a possible increase of the stroke volume. In the present paper, for the sake of simplicity, just one actuation diaphragm is considered.

## 3. The Three-Dimensional Model

Multiphysics modelling encompasses two main parts: the “deformable” solid, containing the actuation portion and the valves for controlling the fluid flux, and the “fluid domain”, which interacts with the solid domain through the interface at the boundary of the solid and fluid domains. Due to the sake of symmetry, half of the micropump is modelled.

**Figure 2:**
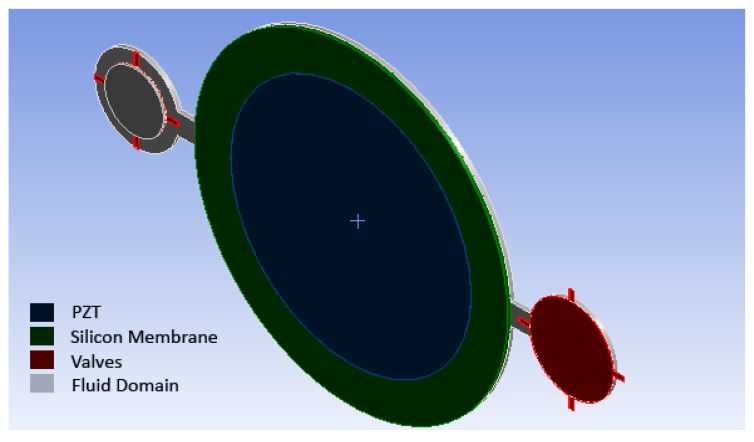
A 3-D scheme of the proposed micropump.

**Figure 3:**
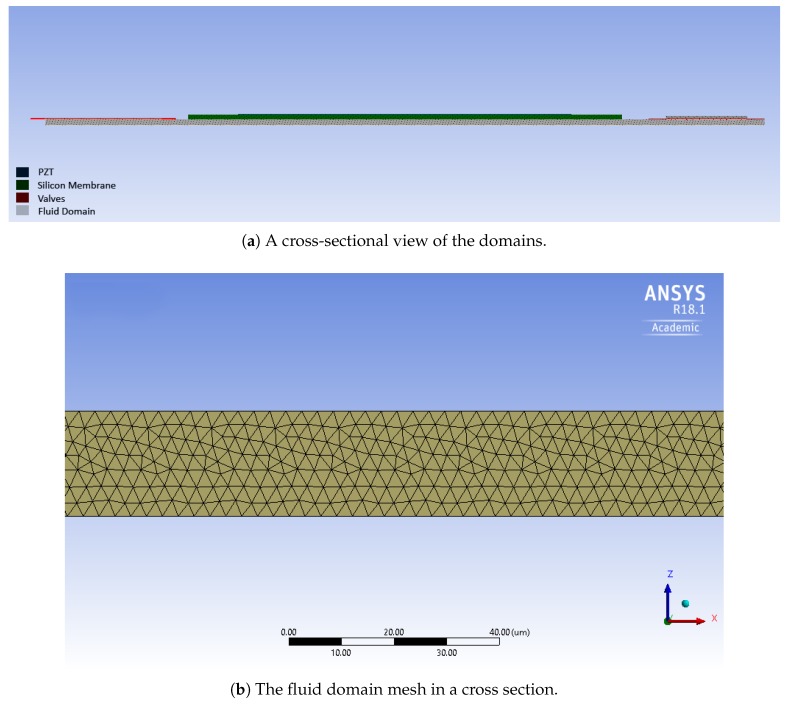
The micropump fluid domain.

In the initial design, the micropump consists of a circular pumping chamber, delimited by lower and lateral fixed surfaces and by an upper silicon diaphragm. As shown in Figure 2 and Figure 3, the fluid domain is completed by two cylindrical spaces from one side connected to the inlet and outlet, which are connected to the pumping chamber by means of two prismatic channels with a rectangular cross section from the other side. It is worth noting that the inlet and outlet are closed by the silicon valves. The radius of the valve’s membrane is larger than the hole, so that the fluid flow is prevented when the valve is in the rest configuration.

The geometrical specifications of the fluid domain are presented in Table 1. The considered fluid is water, modeled as an incompressible, viscid fluid with the mechanical properties summarized in Table 2.

**Figure 4:**
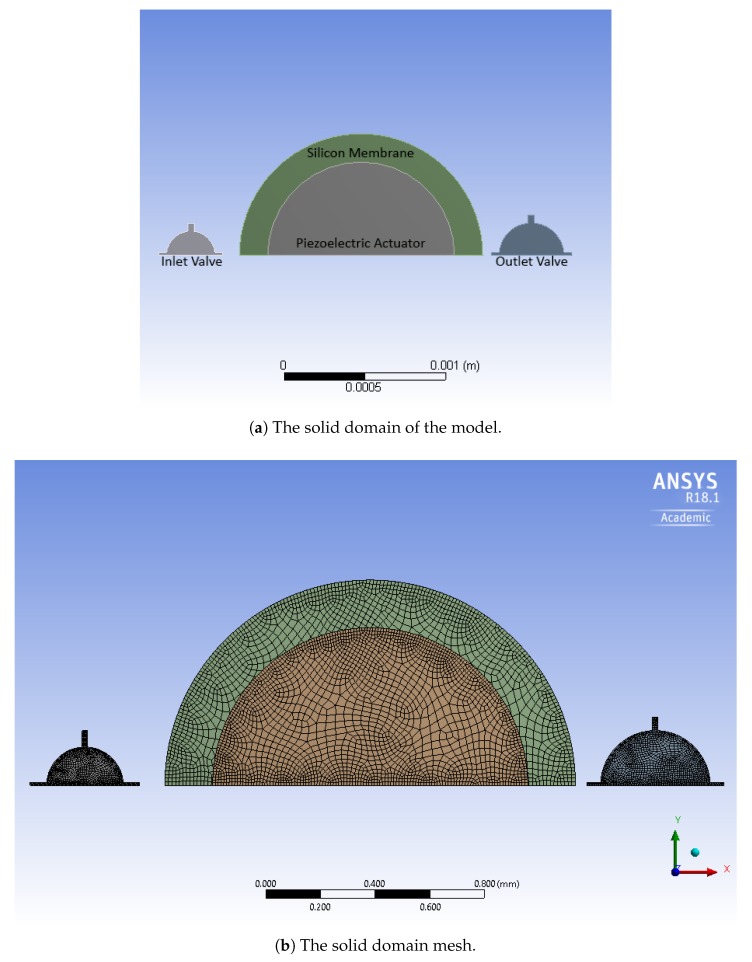
The micropump structural parts.

The model for the solid parts is depicted in Figure 4. The actuation diaphragm is represented by a suspended disc, with a laminate cross section. The actuation is achieved by means of a piezoelectric layer deposited on a silicon plate. Among the various possibilities, lead zirconate titanate (PZT) is chosen as the active material, in the form of a thin film [13]. This is fully compatible with the MEMS production process, through the adoption of one of the available techniques, e.g., sputtering [14], pulsed laser deposition [15], and sol-gel process [16]. The piezoelectric layer has a circular shape, coaxial with the silicon disc. The radius of PZT, see Table 3, is selected according to the results presented in a previous study [6], which showed that the highest stroke volume was obtained when the ratio between the radius of the active layer and the radius of the silicon diaphragm was equal to 0.73. The passive valves consist of silicon discs, that are attached to the rigid frame by means of four rectangular beams. The elastic deformation of the beams allows for the vertical movement of the disc that alternatively opens and closes the inlet hole (the same applies to the outlet). The sealing of the valves in the closed rest configuration is ensured by a prestress in the elastic ligaments, given by an imposed transverse displacement on the edge of the disc.

The mechanical properties of polycrystalline silicon and PZT are given in Table 2.

The investigation of the interaction between the moving solid parts and the fluid is a key point, especially in mechanical micropumps. The motion of the solid, i.e., the oscillating displacement field, induces the motion in the fluid. Also, the fluctuating pressure in the fluid acts as a surface load on the fluid/solid interface. By coupling the governing equations of these two domains, the mutual interaction can be thoroughly studied.

For the proposed micropump, a two-way fluid–Structure Interaction (FSI) is modeled with the commercial code ANSYS^®^ 18.1 and 19.2, with the mechanical and CFX solvers. At each staggered loop, the mechanical solver sends the time derivative of the displacement of the interface nodes to the target nodes in the fluid domain. On the other hand, the CFX solver sends back the stress to the solid nodes based on the traction equilibrium at the interface [17].

The valves are modelled by means of quadrilateral 8-node solid elements (SHELL182 in ANSYS nomenclature), which are suitable for modelling thin to moderately thick shell structures. The aim of choosing this kind of element is to reduce the computational cost, taking into account the specific geometrical features of the problem at hand. The 20-node brick element is chosen for the oscillating membrane (SOLID186), and the coupled-field element is chosen for the piezoelectric actuator (SOLID226). In the fluid domain, the tetrahedral elements with 10 nodes (FLUID221) are used. The number of elements for the solid and fluid domains are 33,968 and 1,425,377, respectively. Some views of the adopted mesh are reported in Figure 3 and Figure 4. The piezoelectric actuator is polarized in the out-of-plane direction. The oscillating membrane is clamped at the edge, as well as the valves at the end of connected bars. The applied voltages to the actuator are 20, 40, and 60 V.

For the boundary conditions on the fluid domain, both the inlet and outlet have a zero relative pressure with respect to the outside of the domain. The faces attached to the silicon membrane and valves are set to the fluid–structure interaction sites as well. The other faces of the fluid domain are set to stationary walls, i.e., the velocity of the fluid is equal to zero.

## 4. Preliminary Results

**Figure 5:**
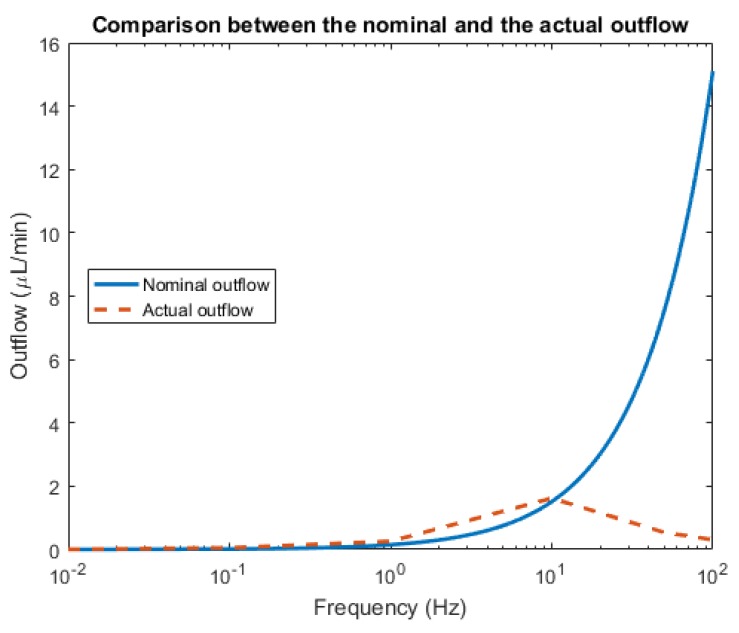
The dependency of the outflow on the pumping frequency.

The proposed micropump is investigated for different values of the input voltage frequency. The most important figure of merit is the outflow of the micropump that is reported in Figure 5 with the actuation voltage equal to 60 V for the range of frequency between 0.01 to 100 Hz. Ideally, the displaced quantity of fluid can be estimated on the basis of the so-called *stroke volume* $v_{s}$, i.e., the variation in the volume of the pumping chamber due to the movement of the actuating diaphragm during one cycle. For a given pumping frequency $f_{p}$, the nominal outflow would be

(1) $$q=f_{p}v_{s}$$

As it is shown by the chart in Figure 5, the computed outflow is strongly different with respect to the nominal quantity. More specifically, the non-monotonic behavior is connected to the inertial force of the fluid so that the maximum outflow is reached at a certain point and a decay is observed after that frequency. The optimal outflow for the designed micropump is achieved at 10 Hz: The numerical results are now thoroughly examined for that specific actuation frequency.

**Figure 6:**
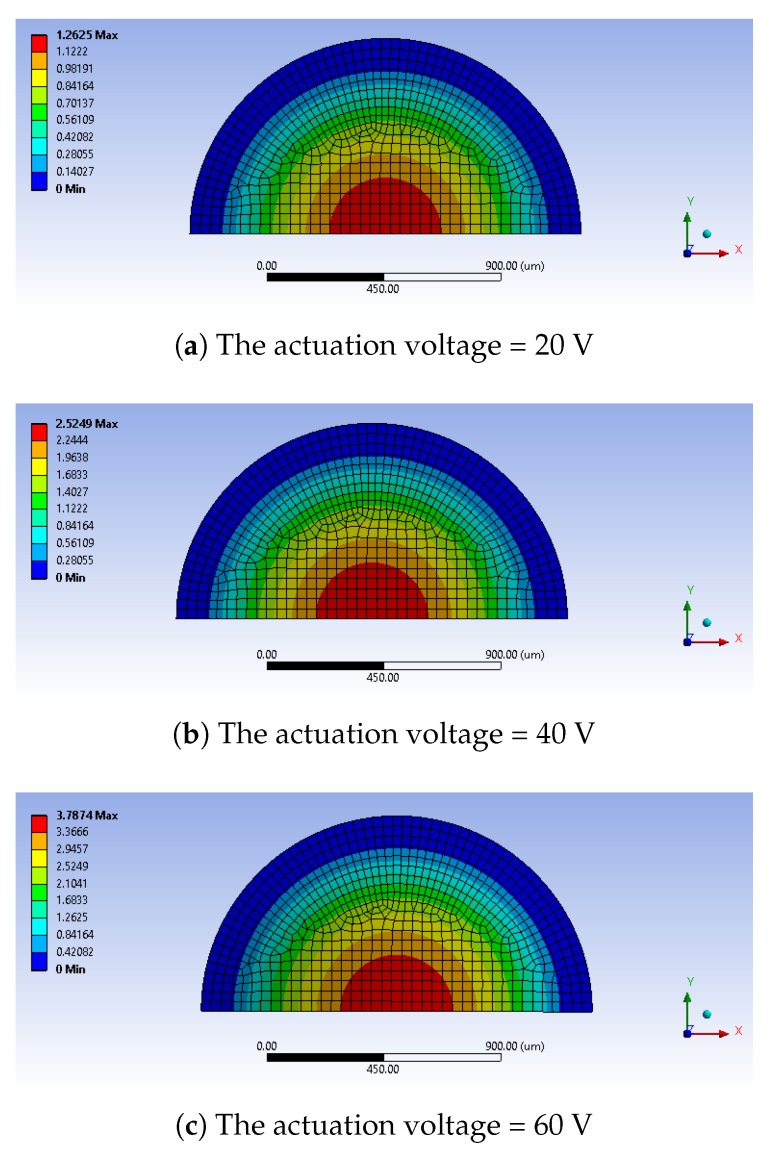
The deformation of the pumping diaphragm: contour plots of the transverse displacement in μm.

At the first stage of the micropump working cycle, the silicon membrane is deformed in the upward direction due to the PZT deformation. The pressure gradient causes an inlet valve opening and the fluid enters in the chamber. The computed maximum deflection of the diaphragm for different actuation voltages is demonstrated in Figure 6.

**Figure 7:**
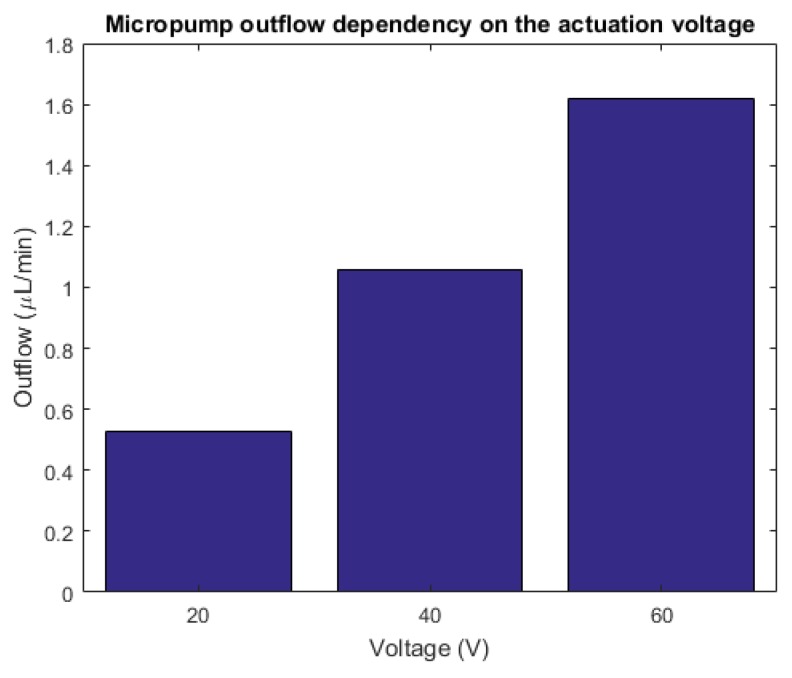
The outflow dependency on the actuation voltage.

Figure 7 represents the outflow of the designed micropump for different actuation voltages at the working frequency of 10 Hz.

**Figure 8:**
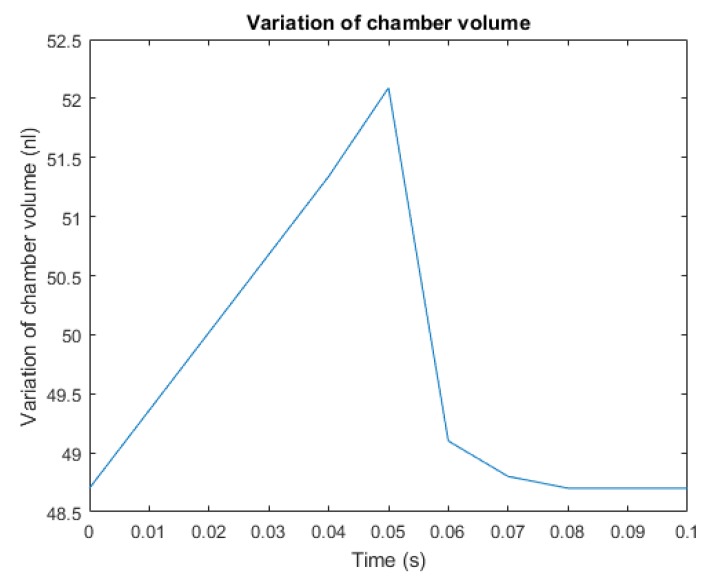
The variation of the chamber volume during a pumping cycle.

Figure 8 presents the variation of the chamber volume during a complete pumping cycle. After the silicon membrane reaches the maximum stroke, as mentioned above, the piezoelectric input is instantaneously dropped and the diaphragm tends to recover its initial configuration. Due to the membrane displacement, the fluid pressure increases, and as a consequence, the outlet valve is pushed in its recess and the hole is opened. At the same time, the elastic recovery enables the closing of the inlet channel. The results of Figure 8 allow one to compute the quantity of fluid that is pushed out from the chamber in a cycle connected to the above defined stroke volume.

**Figure 9:**
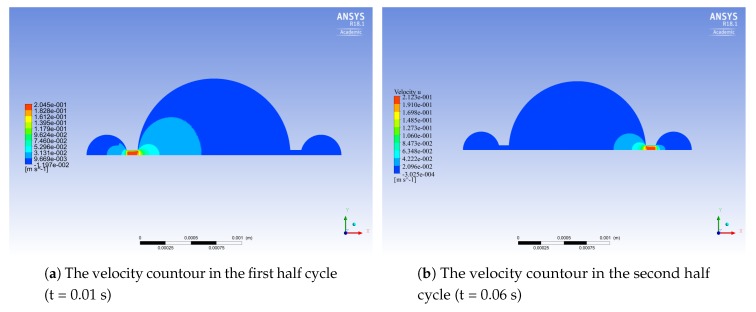
The velocity profiles in the midplane of the micropump.

The analysis shows that the maximum fluid velocity occurs in the channels during the first and the second half cycles (see Figure 9). The maximum backpressure is equal to 6.8 kPa, and the optimal outflow is 1.62 μL $\min^{-1}$.

The apparent power consumption of the actuator can be approximately computed by assuming harmonic variation [18]:

(2) $$P_{A}=I_{e}V_{e}$$

where $I_{e}$ and $V_{e}$ are the root mean square of the current and voltage, respectively. The magnitude of the current generated by the piezoelectric is

(3) $$I=\omega_{p}\int \int D_{3}dxdy$$

where $\omega_{p}$ is the working angular frequency and $D_{3}$ is the electric displacement in the piezoelectric layer. The power consumption for the optimal frequency ($f_{p}$ = 10 Hz) is about 0.29, 0.58, and 0.88 mW for the actuation voltages 20, 40, and 60 V, respectively.

## 5. Geometrical Modifications

### Geometry of the Chamber

**Figure 10:**
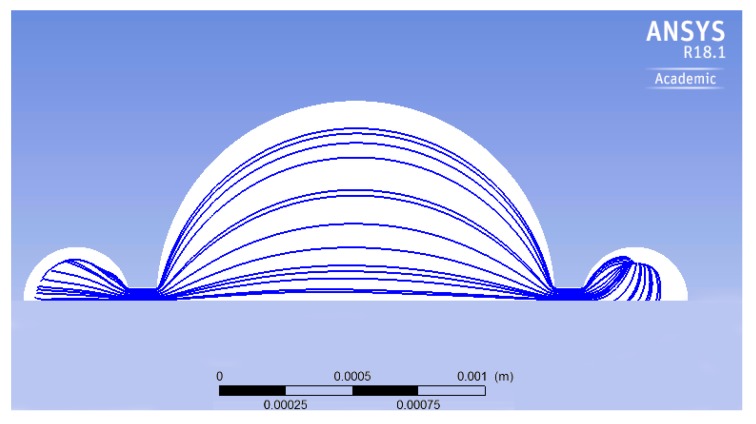
The streamlines of the micropump.

Figure 10 shows the streamlines, during one pumping cycle, within the planar view of the micropump. There are regions close to the perimeter of the pumping chamber where there is no flux. The presence of such regions, called *dead zones*, affects the efficiency of the micropump, since a certain portion of the volume is not involved in the fluid flux. To decrease the dead zones in the pumping chamber, a geometrical optimization is carried out. The specific shape of the streamlines shown in Figure 10 suggests that a better performance is possibly achieved if the circular geometry is replaced by the elliptical one. As a first attempt, the minor axis of the ellipse is kept equal to the original diameter, i.e., 1500 μm, and the major axis is set to 1800 μm. Of course, the shape of the silicon membrane is also changed according to the modification of the chamber geometry. Conversely, the piezoelectric layer remains unchanged, i.e., a circular disc coaxial with respect to the ellipse. The initial volume of the new pumping chamber is 0.0584 mm${}^{3}$, whereas the original volume is 0.0487 mm${}^{3}$ (20% increment).

**Figure 11:**
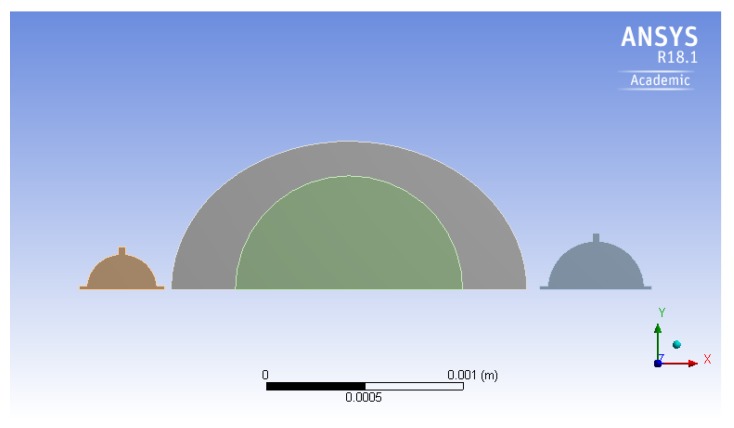
The elliptical chamber micropump.

The modified layout is shown in Figure 11. In this modification, the input voltage remains the same as for the circular geometry: In view of the unchanged geometry of the piezoelectric layer, the power consumption does not change significantly. On the other hand, due to the fact that the stiffness of the elliptical membrane is less than the circular one, the same actuation voltage yields larger deflections of the modified membrane. As a matter of fact, the maximum deflection of the circular case is 3.78 μm, whereas for the elliptical case, one obtains 4.57 μm, a 21% increment.

**Figure 12:**
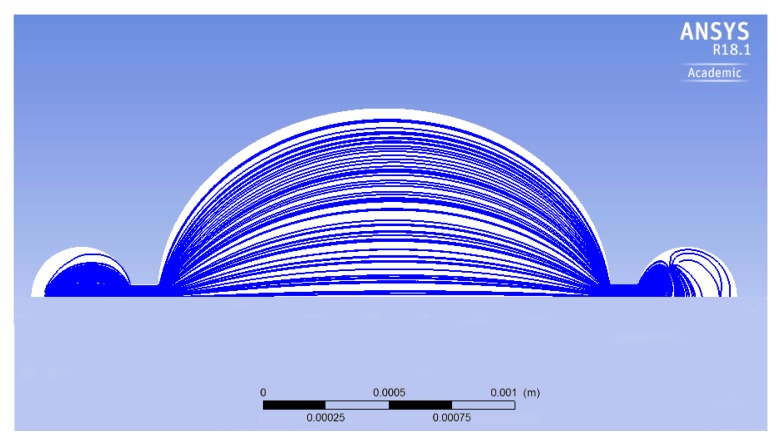
The streamlines in the elliptical geometry.

Figure 12 shows the streamlines for the elliptical chamber: By a comparison with the circular one, it is easily realized that the dead zone is reduced. The obtained outflow of this optimized micropump is 2.11 μL $\min^{-1}$, which indicates that the outflow increases 30%, with the same actuation system and expended power.

**Figure 13:**
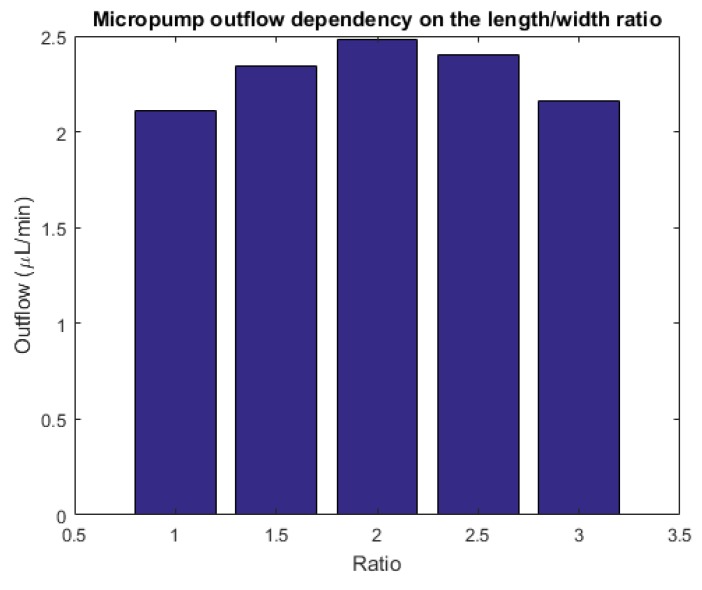
The outflow of the micropump for different length-to-width aspect ratios.

The same analysis has been done on similar geometries with different length/width ratios of the ellipse. As noticed in Figure 13, the numerical outcomes indicate a non-monotonic behavior. For aspect ratios close to unity, the outflow steadily increases in view of the optimized flux and of the larger deflection of the diaphragm. Nevertheless, there is a negative effect of the larger volume of fluid to be displaced. As a consequence, after a certain value, the actuator does not provide sufficient power to promote the fluid motion and the outflow decreases. The optimal outflow is achieved for an aspect ratio equal to 2 (namely, length: 3000 μm, width: 1500 μm); in that case, the outflow attains a value of 2.5 μL $\min^{-1}$.

**Figure 14:**
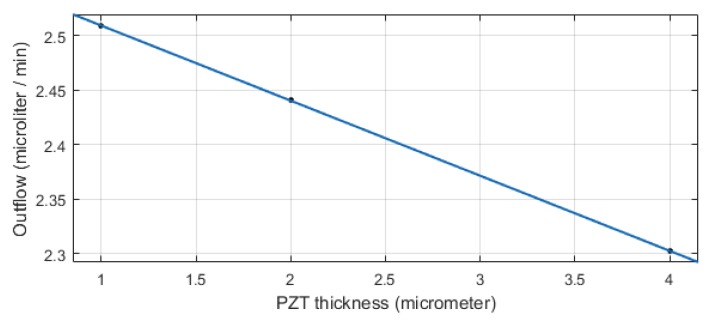
The outflow dependance of the device on the thickness of the lead zirconate titanate (PZT) layer.

In the manufacturing process of this two-wafer micropump, wafer bonding is one important step. There are several methods for silicon wafer bonding such as anodic bonding [19], metal bonding [20], and glass frit bonding [21]. The residual stresses and possible residual warping coming from the bonding process and due to the coefficients of thermal expansion mismatch can have an influence on the mechanical properties of the micropump components and must be carefully controlled. Another important parameter in the fabrication process which affects the performance of the micropump is the piezoelectric layer thickness. The simulations have shown that, by increasing the piezoelectric layer thickness, the outflow decreases linearly (see Figure 14).

## 6. Comparison with a Commercial, Three-Wafer Micropump

**Figure 15:**
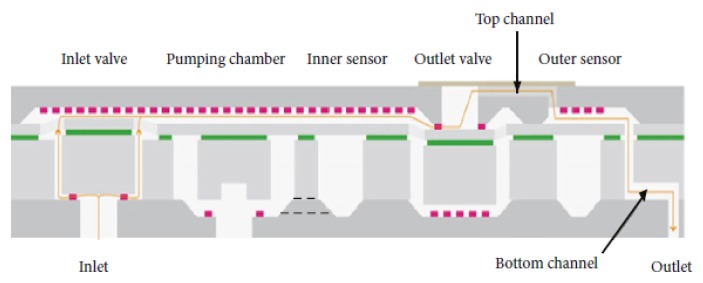
The cross-sectional scheme of the micropump proposed in Reference [22] (Reproduced under Creative Commons Attribution License).

**Figure 16:**
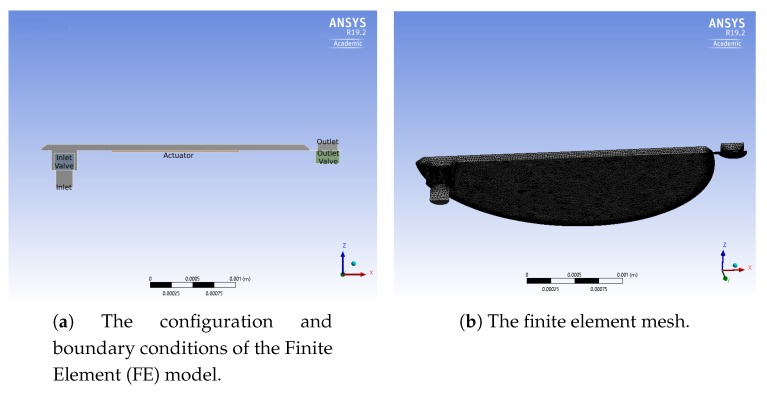
The FE model built to simulate the micropump [22].

To validate the model, a commercial micropump designed for biomedical applications and fabricated by Debiotech [22] is modelled and compared with the proposed new device. The device proposed in Reference [22] is actuated with a bulk piezoelectric actuator, and has passive valves at the inlet and outlet channels. Figure 15 shows the cross section of the device, while Figure 16 presents the FE mesh.

**Figure 17:**
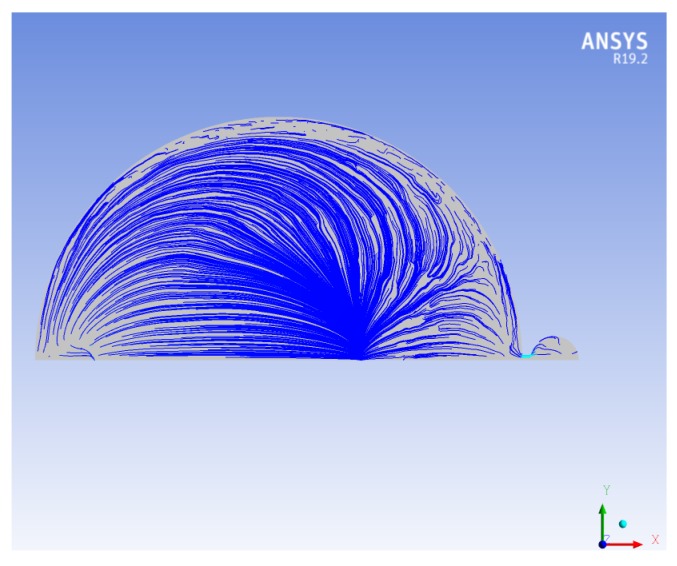
Computed streamlines for the micropump [22] during the pushing phase.

**Figure 18:**
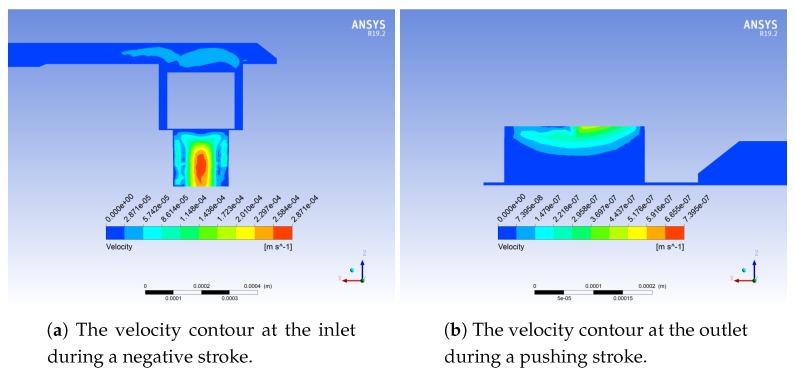
Computed velocity contours at the inlet and outlet of the micropump [22].

A complete pumping cycle at the frequency of 1 Hz is simulated. Figure 17 and Figure 18 show the streamlines and velocity contours, respectively.

**Figure 19:**
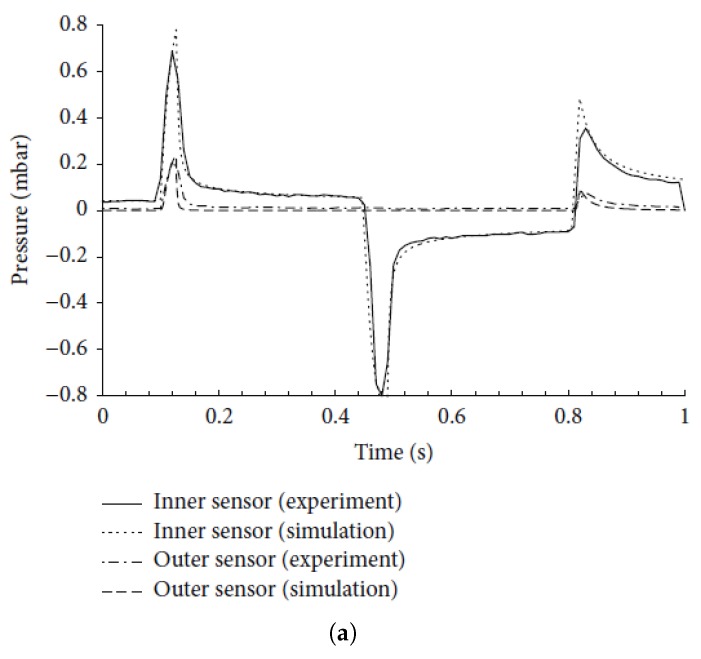

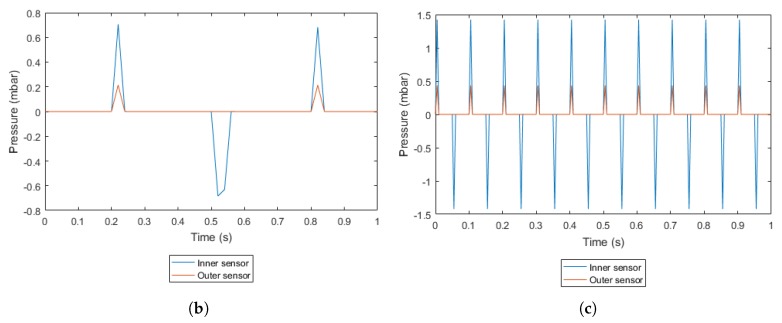
A comparison of the pressure profiles for the micropump [23]: The results for the (**a**) experimental, (**b**) numerical, and (**c**) new device proposed in the present paper. (**a**) The experimental results of the inner pressure sensor. From [23] (Reproduced under Creative Commons Attribution License; (**b**) The pressure profile obtained from the simulation; (**c**) The pressure profile obtained for the proposed device.

To validate the model, the pressure profiles measured inside and outside the chamber in the Debiotech micropump is compared with the results of the numerical model and shown in Figure 19. In addition, the pressure profile obtained at a 10 Hz frequency of the new device proposed in this paper is shown in Figure 19c in the same time window. The numerical model built in the present work for the simulation of the Debiotech micropump has been obtained with the same approach used for the simulation of the new two-wafers piezoelectric micropump proposed in this paper. As shown from Figure 19, the model shows a good agreement with the experimental and numerical results reported in Reference [22]. Note that the pressure peak for the newly designed micropump is 1.5 mbar, which is almost twice that of the Debiotech device.

## 7. Conclusions

In this paper, the response of a new piezoelectric micropump is investigated by means of a multiphysics computational model. The most important novelty of the proposed design, inspired by a recent patent [12] related to electrostatic actuation, is that the device can be manufactured on two silicon wafers only. In that way, important benefits are introduced with respect to existing devices, such as the simplicity of the process and the absence of a multi-stack wafer bonding and its related defects like misalignments between wafers or damages to the middle wafers during the process. The computational outcomes confirm that the proposed micropump can provide a sufficient outflow for biomedical applications that is in the order of 10 μL $\min^{-1}$ [24]. The power consumption appears to be sufficiently limited. The performances of the micropump are boosted by means of simple geometric modifications. By replacing the circular chamber with an elliptical one, the dead zones are decreased significantly and the outflow increases. The analyses suggest that the optimal results are achieved for an aspect ratio equal to 2. Moreover, the presence of rectifiers instead of straight channels is proposed in order to reduce the backflow of the fluid.

Table 4 shows a comparison between the proposed micropump in this research with other devices that have been fabricated up to now. The comparison is satisfactory: The present study paves the way for the development of micropump prototypes that could be tested in order to double check the performance of the new device.

## Figures and Tables

**Table 1:** The geometrical specifications of the fluid domain.

Section	Geometrical Specifications			
	Radius (μm)	Width (μm)	Length (μm)	Thickness (μm)
Chamber	750	-	-	20
Entrance/Exit Cylinders	200	-	-	20
Inlet	140	-	-
Outlet	250	-	-
Channels	-	100	100	20

**Table 2:** The material properties.

Properties	Materials		
	Silicon	PZT-5A	Water
*E* (MPa)	169,000	70,000	-
ν	0.23	0.3	-
ρ (kg/m${}^{3}$)	2300	7700	999.97
$e_{13}$ (N/V m)	-	−5.04	-
$e_{33}$ (N/V m)	-	19.7	-
$\varepsilon_{11}^{s}/\varepsilon_{0}$	-	1320	-
$\varepsilon_{33}^{s}/\varepsilon_{0}$	-	1250	-
Viscosity (cP)	-	-	1 (20 ${}^{\circ }$C)

**Table 3:** The geometrical specifications of the solid parts.

Part	Geometrical Specifications	
	Radius (μm)	Thickness (μm)
Membrane	750	16
Piezoelectric	547.5	1
Inlet Valve	150	2
Outlet Valve	250	2

**Table 4:** A comparison of the proposed micropump with some previously fabricated devices. (${}^{*}$ not reported).

Author and Year	Package Size (mm${}^{3}$)	Frequency (Hz)	Max. Back Pressure (kPa)	ΔV (V)	*q* (μL min${}^{-1}$)
Proposed micropump	8	10	10.2	60	2.5
Van Linten 1988 [25]	4100	0.1	24	NR ${}^{*}$	0.6
Esashi 1989 [26]	800	30	6.4	90	15
Shoji 1990 [27]	4000	50	NR	100	22
Matsumuto 1999 [28]	NR	5	NR	NR	5.5
Li et al. 2000 [29]	4600	20	35	450	2500
MIP Implantable 2003 [30]	357	0.2	55	150	1.7
Liu et al. 2010 [31]	1413	200	22	36	747.6
Luo et al. 2014 [32]	628	120	NR	60	15030
Debiotech 2014 [22]	84	3.125	NR	200	41.67
